# Comparative analysis of image quality and diagnostic performance among SS-EPI, MS-EPI, and rFOV DWI in bladder cancer

**DOI:** 10.1007/s11604-024-01694-1

**Published:** 2024-11-16

**Authors:** Mitsuru Takeuchi, Atsushi Higaki, Yuichi Kojima, Kentaro Ono, Takuma Maruhisa, Takatoshi Yokoyama, Hiroyuki Watanabe, Akira Yamamoto, Tsutomu Tamada

**Affiliations:** 1 Department of Radiology, Radiolonet Tokai, Nagoya, Japan; 2 Department of Radiology, Kawasaki Medical School, 577 Matsushima, Kurashiki, Okayama 701-0192 Japan

**Keywords:** Diffusion weighted imaging, Single-shot echo-planar imaging, Multi-shot echo-planar imaging, Reduced field-of-view, Bladder cancer, Image quality

## Abstract

**Purpose:**

To compare image quality and diagnostic performance among SS-EPI diffusion weighted imaging (DWI), multi-shot (MS) EPI DWI, and reduced field-of-view (rFOV) DWI for muscle-invasive bladder cancer (MIBC).

**Materials and methods:**

This retrospective study included 73 patients with bladder cancer who underwent multiparametric MRI in our referral center between August 2020 and February 2023. Qualitative image assessment was performed in 73; and quantitative assessment was performed in 66 patients with maximum lesion diameter > 10 mm. The diagnostic performance of the imaging finding of muscle invasion was evaluated in 47 patients with pathological confirmation of MIBC. T2-weighted imaging, SS-EPI DWI, MS-EPI DWI, rFOV DWI, and dynamic contrast-enhanced imaging were acquired with 3 T-MRI. Qualitative image assessment was performed by three readers who rated anatomical distortion, clarity of bladder wall, and lesion conspicuity using a four-point scale. Quantitative assessment included calculation of SNR and CNR, and grading of the presence of muscle layer invasion according to the VI-RADS diagnostic criteria. Wilcoxon matched pairs signed rank test was used to compare qualitative and quantitative image quality. McNemar test and receiver-operating characteristic analysis were used to compare diagnostic performance.

**Results:**

Anatomical distortion was less in MS-EPI DWI, rFOV DWI, and SS-EPI DWI, in that order with significant difference. Clarity of bladder wall was greater for MS-EPI DWI, SS-EPI DWI, and rFOV DWI, in that order. There were significant differences between any two combinations of the three DWI types, except between SS-EPI DWI and MS-EPI in Reader 1. Lesion conspicuity, diagnostic performance, SNR and CNR were not significantly different among the three DWI types.

**Conclusions:**

Among the three DWI sequences evaluated, MS-EPI DWI showed the least anatomical distortion and superior bladder wall delineation but no improvement in diagnostic performance for MIBC. MS-EPI DWI may be considered for additional imaging if SS-EPI DWI is of poor quality.

## Introduction

Bladder cancer is the 10th most commonly diagnosed cancer worldwide, with approximately 573,000 new cases and 213,000 deaths annually [1], and is classified into non-muscle-invasive bladder cancer (NMIBC) and muscle-invasive bladder cancer (MIBC) according to the depth of cancer invasion. In general, NMIBC is treated by trans-urethral resection of bladder tumor (TURBT) with bladder sparing, whereas MIBC is treated by radical cystectomy and systemic chemotherapy or radiation therapy [2]. The definitive diagnosis of muscle layer invasive cancer is currently made by pathological examination of tissue resected by TURBT. However, it is a potential problem that the pathological diagnosis of MIBC can be underestimated or overestimated [3–5]. Magnetic resonance imaging (MRI) is used as an adjunct diagnostic tool for MIBC prior to TURBT. The Vesical Imaging-Reporting And Data System (VI-RADS), a standard diagnostic method for MRI of the bladder, was published in 2018 [6]. The standard VI-RADS protocol includes T2-weighted imaging (T2WI), diffusion weighted imaging (DWI), and dynamic contrast-enhanced imaging, with DWI playing a dominant role in the diagnosis of MIBC. DWI is also used to assess the biological behavior of bladder cancer and treatment efficacy [7–9].

Single-shot echo planar imaging (SS-EPI), which is resistant to body motion and has a short acquisition time, is a widely used DWI technique for pelvic organs [6, 10–12]. However, SS-EPI has the weaknesses of susceptibility artifacts due to inhomogeneity of the magnetic field, low spatial resolution, and geometric distortion, which can reduce the accuracy of bladder cancer detection and local staging [13, 14]. New imaging techniques such as multi-shot-EPI DWI (MS-EPI DWI) and DWI with reduced field-of-view (rFOV DWI) are less prone to these problems [15–20]. However, it is currently unclear whether rFOV DWI or MS-EPI DWI outperform SS-EPI DWI for the assessment of bladder cancer. We hypothesized that either rFOV DWI or MS-EPI DWI is better at imaging bladder cancer. The purpose of this study was to compare the image quality and diagnostic performance of SS-EPI DWI, MS-EPI DWI, and rFOV DWI in MIBC.

## Materials and methods

### Patient selection

**Fig. 1:**
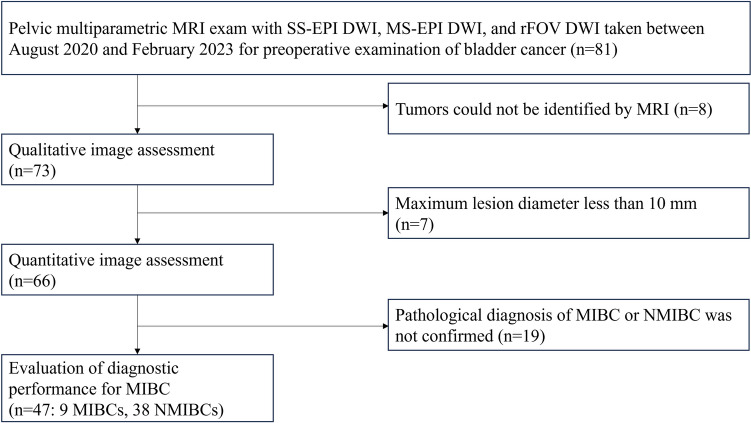
Flow chart of patient selection. *SS-EPI DWI* single-shot echo planar imaging diffusion weighted imaging, *MS-EPI DWI* multi-shot echo planar imaging diffusion weighted imaging, *rFOV-DWI* reduced field-of-view diffusion weighted imaging, *MIBC* muscle-invasive bladder cancer, *NMIBC* non-muscle invasive bladder cancer

Included in the study were 81 pelvic multiparametric MRI scans performed for preoperative examination of bladder tumors between August 2020 and February 2023, in which all of SS-EPI DWI, MS-EPI DWI, and rFOV DWI were acquired. After excluding the scans of 8/81 patients in which bladder cancer could not be identified by MRI, 73 patients (female, 21; male, 52; average age, 74 years) were included in the qualitative evaluation of image quality. In quantitative evaluation of image quality, 7/73 patients with maximum lesion diameter < 10 mm were excluded to minimize measurement error due to partial volume effect, and the images of 66 patients were included. Of these 66 patients, a further were 19 excluded in which the presence or absence of invasion into the muscle layer could not be confirmed pathologically, and 48 patients were included in the evaluation of diagnostic performance of MIBC (Fig. 1). The cohort included 41 participants who were included in a previous study that evaluated the diagnostic significance of peritumoral enhancement in MIBC (being submitted) but not MS-EPI DWI or rFOV DWI.

### Imaging protocol

MRI scanning was performed using a 3.0 T system (Ingenia Elition 3.0 T; Philips Healthcare, Best, the Netherlands) with a 32-channel phased array coil. Intramuscular scopolamine butylbromide or glucagon was administered immediately before the examination to reduce intestinal peristalsis. Patients were not instructed to drink water before the test or to abstain from urinating for 1–2 h, but were instructed to refrain from urinating immediately before the test. Turbo spin-echo T2WI, SS-EPI DWI, MS-EPI DWI and rFOV DWI were acquired in the transverse plane, with sagittal or coronal T2WI added as needed. Three DWI sequences were compared for the purpose of this study: classical DWI with SS-EPI and full FOV, MS-EPI DWI with full FOV, and DWI with rFOV. In MS-EPI, image reconstruction was performed with image-space sampling function (IRIS) [21]. rFOV DWI was scanned with ZOOMed DWI, in which the 180° refocusing pulse is applied obliquely to the 90° slice excitation pulse and outer volume suppression is employed. This technique reduces the FOV and suppresses artifacts caused by peristalsis of the intestinal tract outside the FOV [22, 23]. All DWI sequences used *b* values of 0 and 1000 s/mm^2^. Dynamic contrast-enhanced imaging was acquired in the transverse plane using a 3D T1-weighted gradient echo sequence with fat suppression. After acquisition of pre-contrast imaging, contrast medium (Magnescope, Guerbet, Tokyo, Japan) was injected using a power injector system at a dose of 0.1 mmol/kg body weight at a rate of 3.0 mL/s, followed by a saline flush. Dynamic contrast-enhanced imaging was acquired 7–8 times every 15 s after contrast administration. Table 1 lists the parameters for each imaging sequence in detail.

**Table 1:** MR imaging parameters

	T2WI	SS-EPI DWI	MS-EPI DWI	rFOV-DWI	Dynamic contrast-enhanced imaging
Acquisition time	1:40	2:06	4:12	3:55	2:02 (14.6 × 8)
TR/TE (msec)	2500/80	6000/70	6000/70	2445/76	4.4/1.5
Matrix size	336 × 329	128 × 154	112 × 108	80 × 48	288 × 186
Field of view (mm)	200 × 200	330 × 330	330 × 330	200 × 120	300 × 244
Spatial resolution (mm)	0.6 × 0.6	2.6 × 2.1	3.0 × 3.1	2.5 × 2.6	1.0 × 1.3
Slice thickness (mm)	4	4	4	2.5	3
Slice gap	0	0	0	0	0
Number of shots		1	2	1	1
EPI factor	–	77	27	61	–
Parallel imaging factor	2	2	2	–	1.4
Number of averages	1	6	6	3	1
*b* values (s/mm^2^)	–	0, 1000	0, 1000	0, 1000	–
Bandwidth	288.4	2464.3	3400.4	2388.9	997.7
MPG pulse	–	Three orthogonal directions	Three orthogonal directions	Three orthogonal directions	–
Fat suppression	–	SPAIR	SPAIR	SPAIR	mDixon

*SS-EPI DWI* single-shot echo planar imaging diffusion weighted imaging, *MS-EPI DWI* multi-shot echo planar imaging diffusion weighted imaging, *rFOV-DWI* reduced field-of-view diffusion weighted imaging, *TR* repetition time, *TE* echo time, *MPG* motion probing gradient, *SPAIR* spectral adiabatic inversion recovery, *mDixon* modified Dixon

### Imaging evaluation

#### Qualitative assessment

Qualitative evaluation was performed independently by three radiologists (Reader 1 with 4 years of experience, Reader 2 with 4 years of experience, and Reader 3 with 20 years of experience in bladder MRI). The readers first evaluated SS-EPI for all cases, followed by MS-EPI and finally rFOV. Anatomical distortion, clarity of bladder wall, and lesion conspicuity were rated using the following four-point scale: anatomical distortion, (1) severe, (2) moderate, (3) mild, (4) absent; clarity of bladder wall, (1) poorly visualized, (2) moderate delineation of bladder wall with blurred margin, (3) good delineation of anatomic structure with a sharp margin, (4) excellent delineation of bladder wall; lesion conspicuity, (1) lesion not recognizable, (2) lesion recognizable as slight signal difference, (3) lesion recognizable as distinct signal difference, (4) lesion recognizable as distinct signal difference with a clear lesion margin [17, 19, 21, 24]. In assessing wall clarity, we additionally investigated the number of cases with a low rating (score of 1 or 2) on SS-EPI DWI that showed distortion of the posterior bladder wall due to rectal gas and the number of these cases that improved to a high rating (score of 3 or 4) on the MS-EPI DWI and rFOV DWI.

#### Quantitative assessment

Quantitative image quality assessment was performed by a radiologist (Reader 1). Measurements were taken for all three types of DWI. One circular ROI was placed manually on a tumor > 10 mm in longest diameter in each case. The largest possible ROI was placed within the tumor, without including the tumor margins to avoid partial volume effects. In the case of a stalked lesion, the ROI was placed to avoid the stalk. A circular ROI was placed manually on the iliopsoas muscle as control. For each patient, the mean signal amplitude of the tumor (*SI*tumor) and its standard deviation (*σ*tumor) were recorded, as well as those of the iliopsoas muscle (*SI*control) and (*σ*control). Signal-to-noise ratio (SNR) and contrast-to-noise ratio (CNR) were calculated using Eqs. (1) and (2), respectively [18]:

1 $${\text{SNR}}=\frac{S{I}_{\text{tumor}}}{{\sigma }_{\text{control}}}$$

2 $${\text{CNR}}=\frac{\left|{\text{SI}}_{\text{tumor}}-{\text{SI}}_{\text{control}}\right|}{\sqrt{{\sigma }_{\text{tumor}}^{2}+{\sigma }_{\text{control}}^{2}}}$$

#### Diagnostic performance

The diagnostic performance of the imaging finding of muscle layer invasion in bladder cancer was independently evaluated by the same three radiologists who performed the qualitative image interpretation. Readings were performed in three sessions: SS-EPI DWI was evaluated in the first session, MS-EPI DWI in the second session, and rFOV DWI in the third session. The presence of muscle layer invasion was graded on a five-point scale (1: highly unlikely present, 2: unlikely present, 3: equivocal, 4: likely present, 5: very likely present) using the VI-RADS diagnostic criteria [6]. Readers were blinded to the clinical information and pathology results. The reference standard was the pathological findings of tissue resected by TURBT. In the case that the first TURBT was insufficient to obtain the tumor and the muscle layer immediately below the tumor, the results of the second TURBT were used as the final reference standard.

### Statistical analysis

JMP 11.0.0 (SAS Institute) was used for statistical analysis. The Wilcoxon matched-pairs signed-rank test was used to compare qualitative and quantitative scores that were not assumed to be normal distributed. Shapiro–Wilk test was used to test the normal distribution of the quantitative variable. Diagnostic performance was evaluated using a cutoff of 3 or higher. McNemar test and receiver-operating characteristic (ROC) analysis were used to compare diagnostic performance. Area under the ROC curve (AUC) values were compared by DeLong test. All tests were two-sided. A *p* < 0.05 was considered to indicate significant difference.

## Results

### Qualitative assessment of image quality

**Fig. 2:**
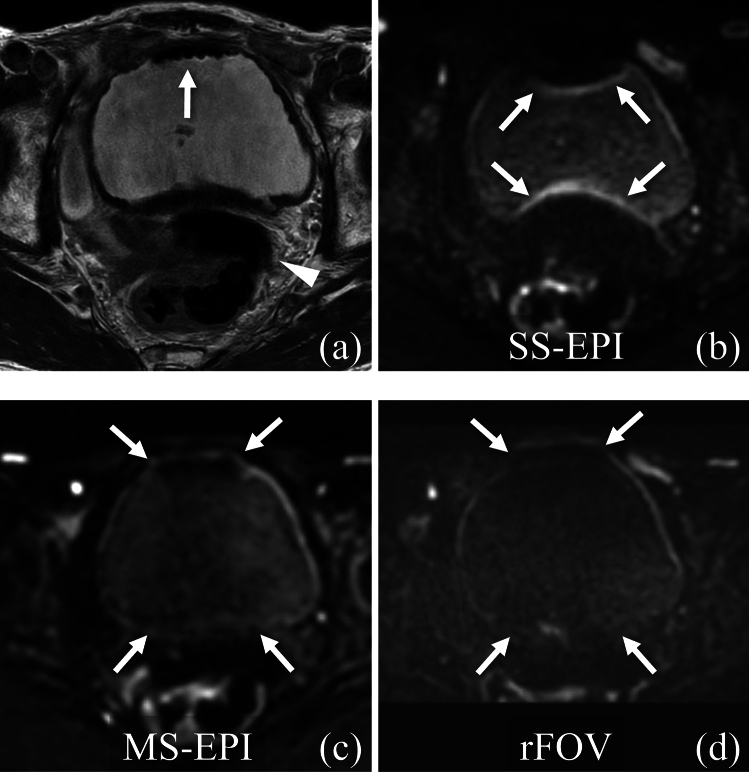
Qualitative evaluation of anatomical distortion. **a** T2-weighted image shows the presence of gas at the ventral side of the bladder (white arrow) and in the rectum (white arrowhead). **b** Single-shot echo planar imaging diffusion weighted imaging (SS-EPI DWI). There is severe anatomical distortion (white arrows) due to gas in the bladder and the rectum. **c** Multi-shot echo planar imaging diffusion weighted imaging (MS-EPI DWI). Although mild anatomic distortion (white arrows) is seen in the anterior and posterior bladder walls, it is markedly improved compared to SS-EPI DWI. **d** Reduced field-of-view diffusion weighted imaging (rFOV DWI). Although moderate anatomic distortion (white arrows) is seen in the anterior and posterior bladder walls, it is an improvement over SS-EPI DWI

**Fig. 3:**
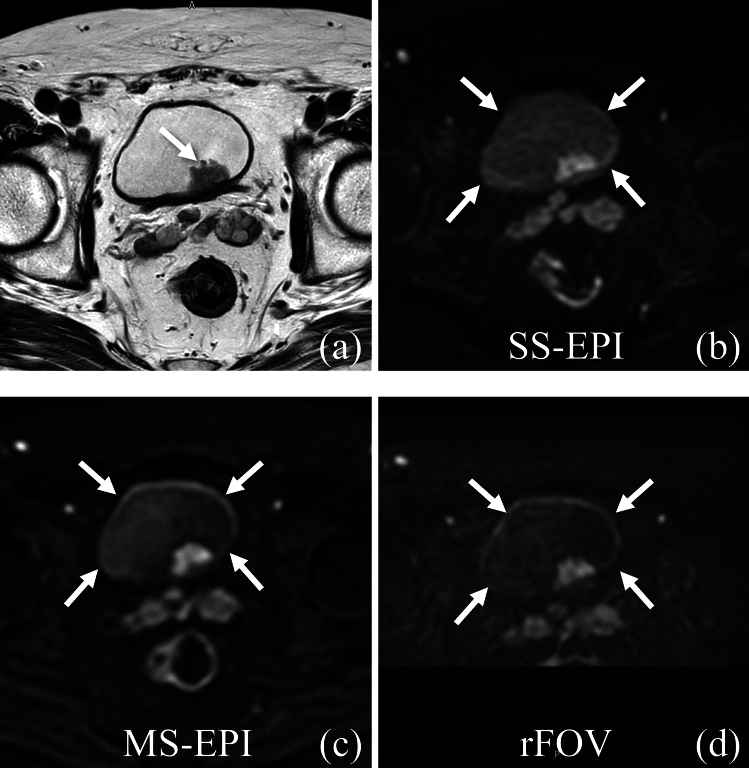
Qualitative evaluation of bladder wall clarity in a 71 year-old man with non-muscle invasive bladder cancer. **a** T2-weighted imaging shows a broad-based tumor (white arrow) at the posterior bladder wall. **b** Single-shot echo planar imaging diffusion weighted imaging (SS-EPI DWI). The bladder wall (white arrows) is poorly visualized. **c** Multi-shot echo planar imaging diffusion weighted imaging (MS-EPI DWI). Visualization of the bladder wall (white arrows) is excellent compared to SS-EPI DWI and reduced field-of-view diffusion weighted imaging (rFOV DWI). **d** rFOV DWI. The bladder wall (white arrows) is moderately delineated with blurred margin but is better than SS-EPI DWI

**Fig. 4:**
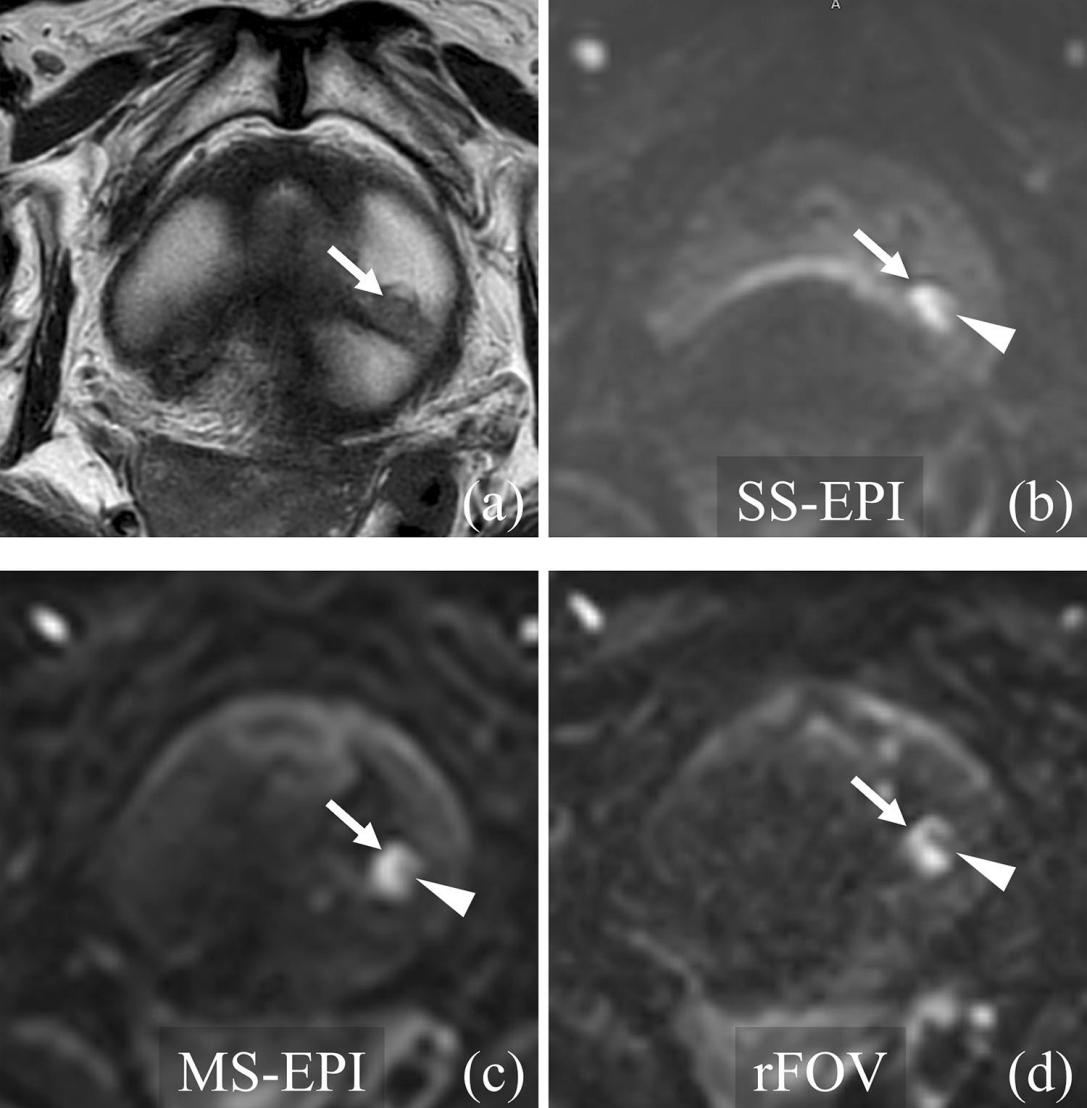
Qualitative evaluation of lesion conspicuity in a 79 year-old man with non-muscle invasive bladder cancer. **a** T2-weighted image shows a tumor (white arrow) protruding from the posterior bladder wall. **b** Single-shot echo planar imaging diffusion weighted imaging (SS-EPI DWI). The tumor (white arrow) is clearly recognizable as an area of high signal, but is slightly deformed and the tumor stalk (white arrowhead) is indistinct. **c** Multi-shot echo planar imaging diffusion weighted imaging (MS-EPI DWI). The tumor (white arrow) is recognizable as an area of high signal with a clear lesion margin and the tumor stalk (white arrowhead) is well defined compared to SS-EPI DWI. **d** Reduced field-of-view diffusion weighted imaging (rFOV DWI). The tumor (white arrow) is recognizable as an area of high signal with a clear lesion margin and the tumor stalk (white arrowhead) is well defined compared to SS-EPI DWI

The results of the qualitative evaluation are presented in Table 2. Anatomical distortion was less in MS-EPI DWI, rFOV DWI, and SS-EPI DWI, in that order, with significant differences between any two combinations of the three types of DWI (*p* < 0.0001–0.0229) (Fig. 2). Clarity of bladder wall was higher for MS-EPI DWI, SS-EPI DWI, and rFOV DWI, in that order (Fig. 3). There were significant differences between any two combinations of the three DWI types (*p* < 0.0001–0.0405), except between SS-EPI DWI and MS-EPI in Reader 1 (*p* = 0.2373). Of the 73 SS-EPI DWI cases for which wall clarity was assessed, 33% (24/73) to 59% (43/73) were rated as 1 or 2 points by the three readers. Of these, 37% (16/59) to 45% (15/45) patients had distortion of the posterior bladder wall on SS-EPI DWI. Of the cases with distortion of the posterior bladder wall, 38% (6/16) to 40% (4/10) had a score of 3 or 4 on MS-EPI DWI and 20% (2/10) to 47% (7/15) had a score of 3 or 4 on rFOV DWI. Lesion conspicuity was not significantly different between the three DWI types in Reader 1 and Reader 2 (*p* = 0.2131–0.7539) (Fig. 4). In Reader 3 alone, lesion conspicuity was significantly higher with MS-EPI DWI than the other two DWIs (*p* < 0.0001–0.0125).

**Table 2:** Qualitative image evaluation

	SS-EPI DWI	MS-EPI DWI	rFOV DWI	SS-EPI DWI vs MS-EPI DWI (*p* value)	MS-EPI DWI vs rFOV DWI (*p* value)	rFOV DWI vs SS-EPI DWI (*p* value)
Anatomical distortion						
Reader 1	2.33 ± 1.31	2.95 ± 1.14	2.74 ± 1.30	< .0001*	0.0009*	< .0001*
Reader 2	3.00 ± 1.37	3.42 ± 1.05	3.18 ± 1.26	< .0001*	0.0013*	0.0229*
Reader 3	2.63 ± 1.14	3.21 ± 0.90	2.92 ± 1.04	< .0001*	0.0001*	0.0006*
Clarity of bladder wall						
Reader 1	3.16 ± 0.5	3.26 ± 0.65	2.86 ± 0.61	0.2373	< .0001*	0.0003*
Reader 2	2.86 ± 0.38	3.00 ± 0.50	2.67 ± 0.47	0.0405*	< .0001*	0.0005*
Reader 3	2.92 ± 0.78	3.34 ± 0.56	2.44 ± 0.58	< .0001*	< .0001*	< .0001*
Lesion conspicuity						
Reader 1	3.66 ± 0.56	3.74 ± 0.55	3.71 ± 0.59	0.2131	0.7539	0.5099
Reader 2	3.96 ± 0.20	3.88 ± 0.44	3.9 ± 0.41	0.2344	0.6875	0.5313
Reader 3	3.37 ± 0.61	3.70 ± 0.54	3.49 ± 0.71	< .0001*	0.0125*	0.1708

Data are presented as the mean ± standard deviation

MS-EPI DWI was the best and superior to rFOV DWI in terms of low anatomical distortion and clarity of the bladder wall; there was little difference in lesion conspicuity among the three DWIs

*SS-EPI DWI* single-shot echo planar imaging diffusion weighted imaging, *MS-EPI DWI* multi-shot echo planar imaging diffusion weighted imaging, *rFOV-DWI* reduced field-of-view diffusion weighted imaging

*p < 0.05, statistically significant

### Quantitative assessment of image quality

The results of the quantitative evaluation are presented in Table 3. There was no significant difference between any two combinations of the three DWI types (*p* = 0.4845–0.6423). There was no significant difference between any two combinations of the three DWI types (*p* = 0.0510–0.5845).

**Table 3:** Quantitative image evaluation

	SS-EPI DWI	MS-EPI DWI	rFOV DWI	SS-EPI DWI vs MS-EPI DWI (*p* value)	MS-EPI DWI vs rFOV DWI (*p* value)	rFOV DWI vs SS-EPI DWI (*p* value)
SNR	50.3 ± 23.9	48.6 ± 18.2	47.5 ± 16.9	0.6423	0.6020	0.4845
CNR	27.7 ± 13.4	28.1 ± 12.0	25.4 ± 14.0	0.5845	0.0510	0.0888

Data are presented as the mean ± standard deviation

Neither SNR nor CNR showed significant differences among the three DWIs

*SS-EPI DWI* single-shot echo planar imaging diffusion weighted imaging, *MS-EPI DWI* multi-shot echo planar imaging diffusion weighted imaging, *rFOV-DWI* reduced field-of-view diffusion weighted imaging, *SNR* signal-to-noise ratio, *CNR* contrast-to-noise ratio

*p < 0.05, statistically significant

### Diagnostic performance

The results of diagnostic performance for NMIBC are presented in Table 4. Of the 47 patients evaluated, 9 were MIBCs and 38 were NMIBCs. Comparison of diagnostic sensitivity, specificity, and accuracy for muscle invasion found no significant difference in any comparison in any reader (*p* = 0.1306–1.0000). AUC values tended to be slightly higher for MS-EPI DWI and rFOV DWI than for SS-EPI DWI for reviewers with fewer years of experience (Reader 1 and Reader 2), but the difference in any combinations of all readers was not significant (*p* = 0.1112–0.9752).

**Table 4:** Diagnostic performance for muscle-invasive bladder cancer by VI-RADS for a cutoff value of category ≥ 3

	SS-EPI DWI	MS-EPI DWI	rFOV DWI	SS-EPI DWI vs MS-EPI DWI (*p* value)	MS-EPI DWI vs rFOV DWI (*p* value)	rFOV DWI vs SS-EPI DWI (*p* value)
Sensitivity						
Reader 1	0.67	0.78	0.89	1.0000	1.0000	0.4795
Reader 2	0.67	0.78	0.78	1.0000	1.0000	1.0000
Reader 3	0.89	0.78	0.89	1.0000	1.0000	1.0000
Specificity						
Reader 1	0.82	0.92	0.89	0.2207	1.0000	0.4497
Reader 2	0.71	0.79	0.84	0.5050	0.6171	0.1824
Reader 3	0.74	0.84	0.79	0.2888	0.6831	0.7518
Accuracy						
Reader 1	0.79	0.89	0.89	0.1306	0.6171	0.1824
Reader 2	0.70	0.79	0.83	0.3865	0.6171	0.1489
Reader 3	0.77	0.83	0.81	0.5050	1.0000	0.7518
AUC						
Reader 1	0.83	0.90	0.93	0.1651	0.5456	0.1112
Reader 2	0.76	0.85	0.83	0.3614	0.5365	0.4406
Reader 3	0.88	0.88	0.91	0.9752	0.4961	0.3774

There were no significant differences in diagnostic performance of muscle-invasive bladder cancer among the three types of DWI

*SS-EPI DWI* single-shot echo planar imaging diffusion weighted imaging, *MS-EPI DWI* multi-shot echo planar imaging diffusion weighted imaging, *rFOV-DWI* reduced field-of-view diffusion weighted imaging, *AUC* area under the receiver-operating characteristic curve

## Discussion

In the present study, we evaluated whether MS-EPI DWI and rFOV DWI can improve image quality of the bladder and bladder tumors and increase the diagnostic performance for muscle layer invasion compared with SS-EPI DWI. To our knowledge, no study has simultaneously compared the three types of DWI, SS-EPI DWI, MS-EPI DWI and rFOV DWI, in the same patient for the evaluation of bladder cancer.

Anatomical distortion due to susceptibility artifact was the least in MS-EPI DWI, which consistent with the results of previous studies in the pelvic area [18, 21, 25, 26]. In the present study, anatomical distortion was less in both MS-EPI DWI and rFOV DWI than in SS-EPI DWI, and was less in MS-EPI DWI than rFOV DWI. MS-EPI DWI employs interleaved k-space trajectories that split the acquisition into many shots, resulting in reduced distortion compared to SS-EPI DWI [21, 27]. The reduced FOV in DWI mitigates the impact of anatomical distortion by confining the region of interest to a limited area, thereby minimizing the influence of external magnetic susceptibility variations. In imaging the bladder, gas in nearby small intestine or rectum or that was introduced into the bladder during urinary drainage was included even in the reduced FOV, which could explain the insufficient reduction of anatomical distortion compared to MS-EPI DWI.

The higher clarity of bladder wall in MS-EPI DWI is probably due to the smaller EPI factor with MS-EPI DWI than the other two DWIs, which reduces distortion and the effects of blurring, consistent with a previous study [18]. In the present study, bladder wall clarity was the lowest for rFOV DWI. In contrast to our results, Juri et al. reported that rFOV DWI delineated the wall more clearly than full-field SS-EPI DWI [17]. However, Juri et al. used a thinner slice thickness for SS-EPI DWI than for rFOV DWI, whereas we used a thinner slice thickness for rFOV DWI and the resulting reduction in SNR might have obscured the wall.

Lesion conspicuity showed no significant variation among the three DWIs except in the case of one observer. Bladder cancer is well-visualized even on SS-EPI DWI, which may have resulted in the lack of discernible difference among the three types of DWI. In fact, there was no significant difference in SNR and CNR among the three DWIs in the present study, consistent with the finding of Chen et al. that readout-segmented EPI DWI and SS-EPI DWI have comparable SNR for bladder cancer [18]. Conversely, several studies have reported higher CNR for MS-EPI DWI and rFOV DWI than for SS-EPI DWI [18, 20]. This may be because CNR is a relative value and is dependent on image parameters such as magnetic field strength, minimum echo time, FOV, matrix, signal average, number of segments, echo spacing, gradient performance, *b* value combination, tissue T2 value, and k-space segmentation direction.

Since VI-RADS specifies DWI as the dominant sequence, DWI imaging with high image quality and low artifacts is required [6]. MS-EPI DWI and rFOV DWI showed no improvement in diagnostic performance over SS-EPI DWI for visualizing muscle layer invasion of bladder cancer. This may be attributable to the absence of significant differences in tumor conspicuity among the three DWI sequences. In addition, diagnostic performance would be unaffected if the tumor was located in an area devoid of distortion artifacts.

Based on these results, MS-EPI DWI had the best image quality overall of the three types of DWIs. However, MS-EPI DWI necessitates twice the imaging time of SS-EPI DWI. Therefore, rather than employing MS-EPI DWI on a routine basis, supplementary MS-EPI DWI should be conducted when distortion artifacts are present on the tumor, or if bladder wall clarity is inadequate on SS-EPI DWI. Even if MS-EPI DWI does not show an overall improvement in diagnostic performance, observations of tumors that are highly affected by artifacts on SS-EPI DWI would be easier to assess on MS-EPI DWI. rFOV DWI may have a lesser role in bladder evaluation because its overall image quality is inferior to MS-EPI and the area it can evaluate is smaller. However, the application of deep learning reconstruction and compressed sensing to DWI has recently been reported, and rFOV DWI has the potential to reduce imaging time and improve image quality and diagnostic performance [28, 29].

The present study has several limitations. The major limitations of the present study were its single-institutional retrospective design. It is desirable to validate the results of this study in a multicenter prospective study. Imaging time, voxel size, and number of averages varied among the three types of DWI. If any one parameter had been unified among the three sequences, the other parameters would not be identical and would also be inappropriate. Therefore, we maintained the parameters for each sequence that were optimized for use at our hospital. The image types of the DWI were not informed to the readers, but because image types differ in appearance, the readers may have been able to identify the image type of the DWI, which may have biased their assessment. The number of cases was not large enough to enable comparison of diagnostic performance in MIBC. Future studies are needed to evaluate the diagnostic performance of MS-EPI DWI and rFOV DWI.

Among the three types of DWI evaluated, MS-EPI DWI showed the least anatomical distortion and superior bladder wall delineation. There was no significant difference among the sequences in terms of diagnostic performance for muscle layer invasion of bladder cancer. MS-EPI DWI rather than rFOV DWI may be considered as an additional sequence in the case of image distortion or obscuration of the bladder wall or tumor on SS-EPI DWI.
